# Third Annual Workshop on Fetal Alcohol Spectrum Disorders Prevention and Clinical Guidelines Research: Supports, Interventions, and Therapies for the Family and Child

**DOI:** 10.1186/s12919-025-00346-0

**Published:** 2025-10-20

**Authors:** Tracey Pérez Koehlmoos, Elizabeth Lee, Ilse Rivera, Jennifer Wisdahl, Tom Donaldson

**Affiliations:** 1 Center for Health Services Research, Uniformed Services University of the Health Sciences, 4301 Jones Bridge Road, Bethesda, MD 20814 USA; 2 Department of Pediatrics, Uniformed Services University of the Health Sciences, 4301 Jones Bridge Road, Bethesda, MD 20814 USA; 3 The Henry M. Jackson Foundation for the Advancement of Military Medicine, 6720 A Rockledge Dr, Bethesda, MD 20817 USA; 4 FASD United, 1054 31st Street NW #204, Washington, DC 20007 USA

## Executive summary

Fetal alcohol spectrum disorders (FASD), a neurodevelopmental disorder, affects an estimated 1 in 20 school-aged children in the United States [1], making it one of the leading preventable causes of developmental disabilities. FASD comes with a range of physical, cognitive, emotional, and social challenges that result from prenatal alcohol exposure. Addressing FASD in the military requires tailored strategies to support individuals and their families, with a focus on prevention, early intervention, and comprehensive care.

The Uniformed Services University of the Health Sciences (USUHS), in conjunction with FASD United, hosted the third annual *Workshop on Fetal Alcohol Spectrum Disorders Prevention and Clinical Guidelines Research: Supports, Interventions, and Therapies for the Family and Child* on 18 September 2024 in Washington, DC. This was to gain an understanding of the current scientific landscape and create care approaches that are grounded in and adaptable to the latest insights on FASD. As part of a four-year, federally funded health services research initiative on FASD in the U.S. Department of Defense (DoD) Military Health System (MHS), the workshop examined the initiative's progress, reviewed current knowledge and practices in research and clinical fields, and identified potential strategies to enhance prevention, screening, diagnosis, interventions, and family support. More than 80 attendees from academia, health care, federal agencies, and patient advocacy organizations met to share research findings and progress updates; learn from living experiences; and discuss ways to advance research, screening, and services for at-risk women and families experiencing FASD.

## Background

The FASD Prevention and Clinical Guidelines Research Initiative is a four-year, federally funded project initiated in 2022 that is focused on FASD in the MHS. As part of the initiative, the Center for Health Services Research (CHSR) at USUHS, FASD United, and the Boston University School of Public Health are working together to examine the experiences of families and providers in the military community who care for and support those individuals living with FASD.

The project, first and foremost, aims to increase awareness of and the response to FASD and its risk factors within MHS. In addition, by strengthening the research basis for FASD prevention and clinical guidelines, researchers and practitioners involved in the project hope to improve care delivery to military children and families and create a new model of FASD care delivery that could be replicated in the civilian sector.

As one of the nation’s largest and most comprehensive healthcare systems, the MHS delivers direct services and private-sector care to approximately 9.6 million beneficiaries, including active-duty service members, military retirees, and their families. CHSR directly supports the DoD and MHS, conducts research aligned with MHS strategic goals, provides Health Services Research (HSR) training for students and faculty, and collaborates with civilian HSR organizations. FASD United is a leading voice for the FASD community that seeks to expand the reach of FASD-informed diagnostic, medical, behavioral health, and non-clinical services while also advocating for policy changes that ensure full inclusion in all care systems and benefits programs for individuals with FASD. FASD United also works to prevent prenatal exposure to alcohol and other harmful substances.

## Introduction

USUHS and FASD United hosted the *3rd Annual Workshop on Fetal Alcohol Spectrum Disorders Prevention and Clinical Guidelines Research: Supports, Interventions, and Therapies for the Family and Child* on 18 September 2024 in Washington, DC. More than 80 attendees from academia, health care, federal agencies, and patient advocacy organizations gathered to review the latest research and discuss key issues surrounding FASD during the day-long workshop (see Appendix A for a list of attendees).

The event was the third in a series of workshops held as part of the FASD Prevention and Clinical Guidelines Research initiative. The first of these annual workshops was held in 2022 and provided an overview of the existing research, programs, and clinical practice guidelines focused on prenatal alcohol exposure and FASD [2]. The second workshop, held in 2023, detailed several lines of effort, including plans for assessments of community needs and population health, the use of electronic health records and predictive analytics to improve early diagnosis and patient outcomes, and a new hub-and-spoke model for telehealth [3]. The 2024 workshop, summarized in this report, focused on ways to advance support, interventions, and therapies for both the family and child.

USUHS’s external partners in this effort are FASD United, Boston University School of Public Health, and the Henry M. Jackson Foundation for the Advancement of Military Medicine. Internal USUHS partners are the Center for Health Services Research (CHSR); the School of Medicine Departments of Preventive Medicine & Biostatistics, Clinical Psychology, Gynecologic Surgery and Obstetrics, Family Medicine, and Pediatrics; and the Graduate School of Nursing.

Tracey Pérez Koehlmoos, Director of the CHSR at USUHS, and Tom Donaldson, FASD United President and CEO, provided welcoming remarks. They expressed optimism that this novel effort could be used to transform the field of FASD, particularly for families, by sharing new research findings to assist the larger neurodevelopmental community. The workshop began with a keynote presentation that provided historical background on FASD, reviewed the status of interventions, and laid out key steps for moving the field and FASD interventions forward. Next, researchers provided updates on various projects and studies related to the FASD Prevention and Clinical Guidelines Research initiative. These research presentations were followed by a panel on living experiences shared by military families impacted by FASD.

The workshop concluded with an interactive working session, during which participants were divided into groups to discuss needs and perspectives related to FASD family support and interventions in health care. The working session report-outs underscored the importance of adopting a comprehensive, informed approach to supporting families impacted by FASD, while highlighting the need for multi-layered strategies that integrate prevention, early diagnosis and intervention, education, and long-term care to effectively address the complex challenges these families face. Throughout the workshop, participants emphasized the dearth of resources and accessibility challenges, the importance of providing FASD education to healthcare providers, developing FASD-informed care and interventions, addressing social stigmas around the condition, and understanding and addressing both the complexities of FASD and the added stressors for military families impacted by FASD.

## Shaping the future of care in the field of FASD

Heather Carmichael Olson, University of Washington School of Medicine and Seattle Children’s Research Institute, set the stage for the workshop discussions with a historical perspective on FASD research and clinical services. She also reviewed some of the latest research on interventions and outlined concrete steps for advancing FASD research and interventions.

### Evolution of FASD awareness and care

Olson reviewed how FASD research, advocacy, and clinical care have evolved over the past 50 years. Physicians and researchers first identified fetal alcohol syndrome (FAS) in the late 1960 s and early 1970 s, spurring the launch of studies in animal models and human populations to understand the effects of prenatal alcohol exposure (PAE). Soon after the scientific community began exploring the topic, family support networks began to form with the goal of developing interventions and advocating for action. Using past pamphlets as examples, Olson showed how outreach and education surrounding FASD began with a focus on awareness and prevention. One positive outcome from the growing FASD awareness was that families, researchers, government organizations, and advocacy groups were all working together. However, she noted that an early focus on the negative aspects of the condition also had the unintended effect of introducing stigma, a challenge that has persisted to this day.

FASD diagnostic clinics, expert insights, and living experience have greatly expanded since the 1970 s, building a wealth of clinical and family knowledge on effective interventions. This accumulated wisdom has guided treatment approaches, even though formal intervention research has developed more slowly. Specialized FASD diagnostic clinics began to emerge in the 1990 s and have gradually expanded in the years since. In 1996, the Institute of Medicine published a report calling for clinical services for people with FASD [4], and by the early 2000 s, momentum was growing for targeted intervention research. During this same period, researchers also identified what they called secondary disabilities — later termed adverse impacts — within FASD [5]. This work highlighted behavioral challenges and life difficulties, but because it focused on the negative, it again unintentionally contributed to stigma, Olson said, creating an additional obstacle for families seeking care and making it difficult to build and sustain hope.

The term FASD replaced FAS (Fetal Alcohol Syndrome) in 2004, and in 2009, the U.S. Centers for Disease Control and Prevention (CDC) issued a call to action as a published report [6]. Expert recommendations were made for what should be done about FASD. Another milestone came in 2013, when the mental health field formally acknowledged FASD by including the term neurobehavioral disorder associated with prenatal alcohol exposure (ND-PAE) in the fifth edition of the Diagnostic and Statistical Manual of Mental Disorders [7]. Following this, the term FASD-informed care came into use, and the research and clinical fields began to see rapid growth in attention to FASD interventions, services, and systematic research to advance FASD-informed systems of care.

### A growing focus on social determinants of health

Interventions for FASD advanced as researchers began to identify protective factors that could mitigate the adverse impacts of the condition. Olson said that a big driver of this work has been the recognition that many issues related to FASD are rooted not within individuals themselves, but in social determinants of health. This recognition spurred calls to action, led to critiques of the existing scientific literature, increased demand for treatment, and renewed advocacy efforts, leading to greater pressure for government agencies to fund FASD research. As a result, this focus on social determinants and mitigating factors has fueled a growing body of systematic treatment research since the early 2000s, deepening understanding of the factors that mitigate adverse impacts and strengthening interventions. At the same time, the incredible diversity of FASD has become clearer, leading to a growing recognition that FASD varies from person to person and across different ages, socioeconomic levels, geographies, and cultures.

Olson pointed out that FASD presents a daunting global public health challenge that is not limited to specific groups or cultures. This worldwide scope unites countries and fosters international efforts to address it collaboratively. It also comes with a responsibility to ensure that interventions are culturally appropriate and that treatments and interventions resonate with those receiving them. To accomplish this, she underscored the importance of listening to the voices of those individuals with FASD. Thanks to advances in qualitative research methods, she noted that it is now possible to gather themes from what people say and use this to empirically describe information learned from living experiences, helping to strengthen the connections between scientific evidence and the FASD community.

### Advancing FASD-informed care

Olson posited that FASD intervention is now at a tipping point, a place where progress is unstoppable. She noted that bringing together the latest research data is essential to this process, and ongoing efforts in meta-analysis and narrative reviews are advancing the goal of consolidating recent insights. As an example, she highlighted the chapter “FASD-informed care and the future of intervention” [8] in the book “Fetal Alcohol Spectrum Disorder: A Multidisciplinary Approach”. This comprehensive chapter includes three narrative reviews that compile relevant theories, living experience data, and published and in-process intervention data specifically gathered from those with FASD or PAE. In addition to the formal scientific literature, she noted that the chapter also explores informal “gray literature” to capture additional insights from living experiences.

**Fig. 1:**
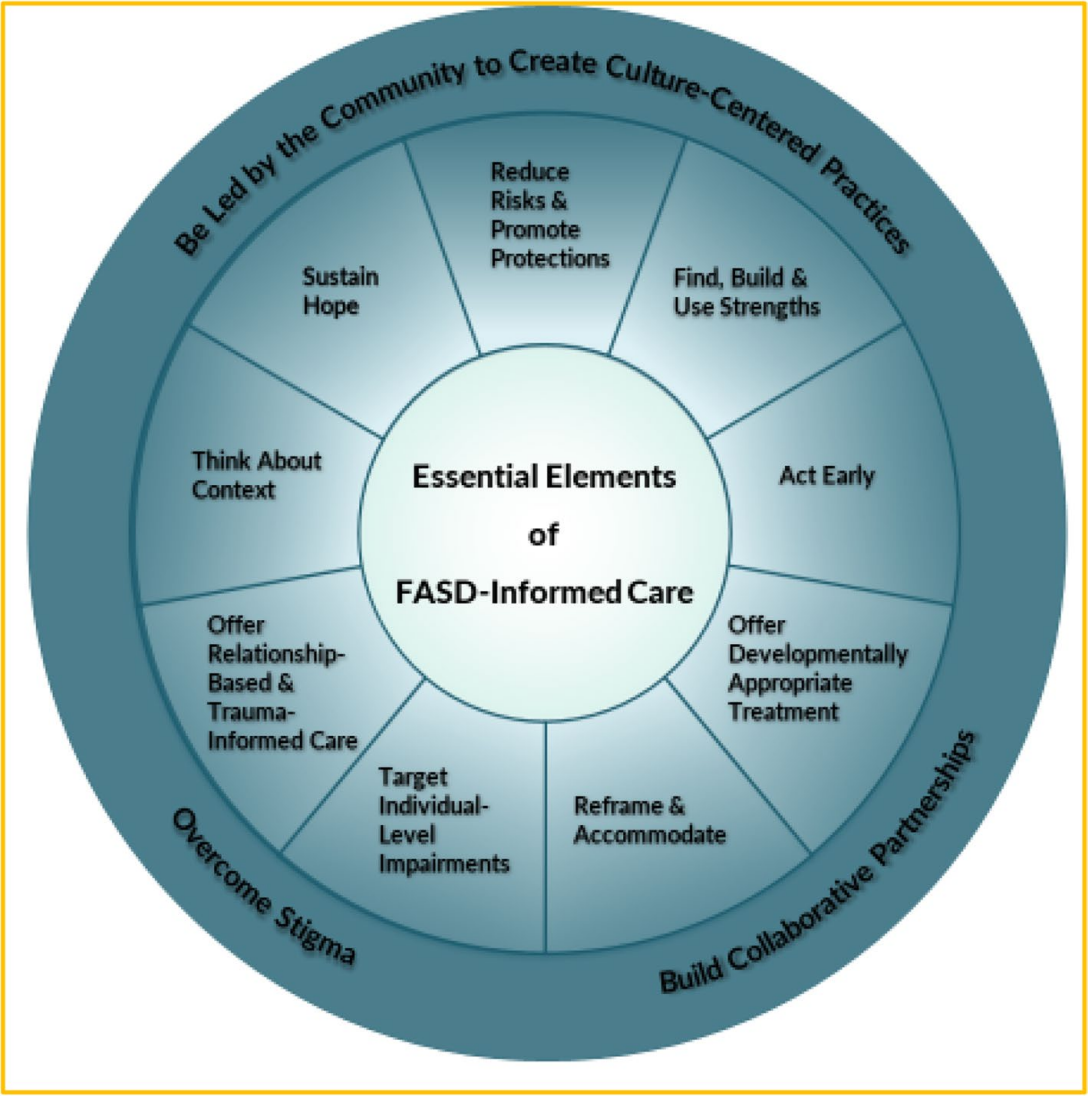
Essential Elements of FASD-Informed Care. Source: Olson HC, Pruner M, Byington N, Jirikowic T. (2023). FASD-Informed Care and the Future of Intervention. In: Abdul-Rahman OA, Petrenko CLM. (eds) Fetal Alcohol Spectrum Disorders. Springer, Cham. 10.1007/978-3-031-32386-7_13. Reprinted with permission from Springer Nature

In the chapter, Olson and her co-authors identified 12 essential elements of FASD-informed care that can be used when developing interventions. These include the three complex overarching elements of overcoming stigma, building collaborative partnerships, and being led by the community to create culture-centered practices. The overarching elements influence the more specific elements of reducing risk and promoting protections; finding, building, and using strengths; acting early; offering developmentally appropriate treatment; reframing and accommodating; targeting individual-level impairments; offering relationship-based and trauma-informed care; thinking about context; and sustaining hope (Fig. 1).

Olson pointed to the FASD United Family Navigator program as an example of an intervention that provides FASD-informed care [9]. One essential element of FASD-informed care is to sustain hope, and Olson described how, when a person speaks with a family navigator, they build and sustain hope because they know that someone knowledgeable is listening. The navigator also puts the other elements of FASD-informed care into practice by focusing on strategies to build protective factors and reduce the risk of later adverse outcomes. For example, this can include preventing traumatic experiences and laying the groundwork for positive school experiences, such as collaborating with schools to develop effective individualized education plans (IEPs). The program also aims to overcome stigma by connecting families with knowledgeable individuals who can provide appropriate language and enhance understanding for those who may be less familiar with the issues. Additionally, it emphasizes the importance of establishing collaborative partnerships with various organizations and agencies. Over the years, the FASD United Navigator Program has developed a clear understanding of what can be done within the program to provide elements of FASD-informed care.

Olson said that an important next step is to measure the Family Navigator program, and other interventions, against the "essential elements" framework of FASD-Informed Care. This can help providers and researchers work toward improving the capacity of interventions to better serve individuals with FASD or PAE and their families.

FASD interventions fall into several categories which focus on different strategies and groups. This was based on the narrative review conducted by Olson and her team, which focuses on published and in-process intervention research available so far, specifically focused on those with FASD or PAE. Relationship-based and multiple-component early interventions target infants and young children. Skill-building treatments, which can be geared toward children from the toddler to the teen years, address areas such as executive function, self-regulation, social communication, attention and memory, academics, sensory-motor skills, and physical activity. Parenting programs, which can support caregivers from the prenatal period through early adulthood, can be grouped into two categories: those that focus on positive parenting strategies, and those that focus on parenting support, education, and advocacy. In research so far, Olson noted that programs targeting adolescents and transition-aged youth are smaller in number and tend to focus on particular populations or specific issues, such as preventing substance use or decreasing other adverse impacts, suggesting a need for further expansion in this area.

### Action steps to continue progress

While many FASD interventions have been tested, Olson lamented that very few are currently being implemented. She commented that it is imperative that FASD interventions for which there is good evidence be disseminated and implemented. But there are other steps that can also be taken. Olson outlined four strategic action steps to move interventions forward and benefit more families: advancing FASD recognition, providing evidence-informed care, offering person-centered planning, and exploring evidence-based treatments.

For the first step, advancing FASD recognition, Olson noted that significant efforts are being made to improve recognition of FASD among clinicians while also addressing and reducing the stigma associated with the condition. For example, the Extension for Community Healthcare Outcomes (ECHO) approach for delivering FASD education to health care providers offers several programs on these topics [10]. In another example, a statewide initiative in Michigan aims to offer FASD screening and education to community mental health providers and centers, as well as supporting interventions through FASD-informed community mental health services.

The second step is to provide basic evidence-informed care. While the work of implementing tailored evidence-based treatment for FASD is moving forward, basic education can be provided so that existing interventions can be informed by the available evidence. Since systems of FASD-informed care are still under development, Olson said that evidence-informed programs like the FASD United Family Navigator program can provide valuable support as evidence-based treatments advance. Another useful resource for supporting evidence-informed care is TIP 58 [11], which was developed by the Substance Abuse and Mental Health Services Administration to provide information on principles and clinical techniques for mental health and substance use treatment settings. The CDC also provides a list of various treatments for FASD, and Olson noted that resources such as the Fetal Alcohol Spectrum Consultation Education and Training Services Neurobehavioral Framework provide information and guidance that is informed by both scientific evidence and living experiences [12, 13].

The third step is person-centered planning, an approach to care that places the person with FASD at the center of the services. With this approach, individualized services are developed collaboratively in partnership with professionals, families, and the person being served. Accomplishing this requires understanding the person's individual needs, their functional profile, and their strengths and challenges from a neurodevelopmental perspective, and then designing school programming, home programming, and community resources that match those needs. Person-centered planning inherently fits well with the individually variable, diverse needs of individuals with FASD. But this method can be improved by bringing the essential elements of FASD-informed care to person-centered planning.

In addition to implementing tailored and/or new evidence-based treatments for FASD, a final action step is exploring existing evidence-based treatments for other clinical populations. Examples include parent-child intervention, cognitive behavioral therapy, or behavioral parent training. Olson said that there is sometimes an assumption that existing evidence-based treatments developed for other conditions do not work for people with FASD, and suggested that this assumption needs to be critically examined using controlled research studies. This type of work is important to determine which treatments are most useful and what aspects of them might need adapting for the FASD community. Olson noted that one example of an evidence-based treatment that worked without adaptations (for a portion of the sample) is an emotion-focused parent-child intervention for families called Tuning in to Kids [14]. Examining this intervention in the FASD context, researchers have found promising evidence that the program could bring positive outcomes for younger parents with no special adaptations.

Overall, Olson emphasized that shaping the future of FASD care will require understanding what treatment adaptations are needed for existing evidence-based treatments (and what features should be built into new treatments) — and then building the evidence base and addressing treatment gaps. As an example, she said that this has been done already for the Alert Program for self-regulation, which has demonstrated behavioral outcomes and neurobiological outcomes in several research projects with various adaptations for individual, group, school, and home contexts [15]. Olson noted that the evidence base is strong enough for this program to be listed in the California Evidence-Based Clearinghouse for Child Welfare, a registry of evidence-based practices relevant for children and families involved in the child welfare system.

Once interventions are developed, Olson emphasized that it is critical to study best practices for taking them out into the community and identify any barriers to using them. Treatments also need to be scalable to be used on a community-wide level. Finally, Olson reiterated that it is key to collaboratively work with the community to ensure that treatments are culture-centered and fit the communities in which they are deployed to support a successful continuum of care.

### Families Moving Forward Program

As an example of a continuum of FASD-informed care, Olson described the Families Moving Forward Program- and options for care that have developed from this foundational intervention. Olson and colleagues first developed, tested, and are offering training for clinicians in multiple U.S. states and in Canada to use a positive parenting intervention called the Families Moving Forward (FMF) Program [16]. Developed at Seattle Children’s, this manualized yet individualized behavioral consultation intervention is tailored for families raising children 3-13 years old with PAE or FASD, to support parental education and coaching for complex family situations where children have concerning behavior problems. Olson described how she then worked with a team led by Christie Petrenko and Cristiano Tapparello at the University of Rochester to make the foundational FMF Program even more accessible and scalable by turning it into a mobile health application called FMF Connect. Led by the University of Rochester, this was then further transformed into a clinician-assisted version called FMF Connect Pro that includes clinician training and is now being tested. A teacher-focused website was also developed, based on the FMF Program and FMF Connect, to provide materials tailored for general and special educators.

The Specialized Neurodevelopmental Assessment and Consultation Service (SNACS) Clinic is another option for care based on the foundational FMF Program. Developed at Seattle Children’s, the SNACS model, which uses 3-5 sessions to deliver a short-term specialized assessment and consultation service, is currently in use at the Seattle Children’s Autism Center and can be reproduced elsewhere. SNACS includes therapeutic assessment, diagnosis, and feedback, often conducted through telehealth, with psychologists creating a customized functional profile for the child and engaging with families on essential FASD-informed care topics. The model has proven feasible and flexible, serving a diverse population and effectively connecting families to child-focused interventions or intensive parent support as the next steps. Based on these factors, Olson suggested that the scalability of SNACS and the FMF model in general could be a valuable addition to the hub-and-spoke framework being developed for supporting military families impacted by FASD.

Thinking about challenges in treatment, Olson posited that a transdiagnostic approach — which applies the same treatment logic and procedures beyond a particular diagnosis — might be appropriate as one lens for advancing FASD-informed care. In light of the history of the FASD field, Olson said that a traditional focus on specific diagnoses can limit understanding of the broader spectrum of issues involved. In real-world scenarios, reliable diagnosis of FASD is often hindered by unknown PAE, unrecognized FASD, and the stigma surrounding these conditions. Olson suggested that adopting a transdiagnostic approach can pave the way for innovative treatments that address not only FASD and PAE, but also the complex learning and behavioral challenges arising from trauma and other prenatal exposures.

## FASD prevention and clinical guidelines research project updates

Five researchers shared emerging findings from programs and projects related to the FASD Prevention and Clinical Guidelines Research initiative. Their presentations were followed by a discussion of cross-cutting issues and themes related to scientific practices and progress on FASD.

### Community needs assessment

Ilse Rivera, CHSR, provided an overview of the FASD Prevention and Clinical Guidelines Research initiative’s five lines of effort as foundational context for more detailed updates on work that is ongoing under the second line of effort. The first line of effort is to map all resources available within the MHS to address FASD, and has been published in *Military Medicine* [17]. The second line of effort is a community needs assessment examining experiences with FASD in the MHS. The third and fourth lines of effort will examine the burden of PAE, FASD, and related conditions to create a model for health care pathways in the MHS. The fifth line of effort aims to create a tele-education and telehealth hub and spoke model across the MHS.

Focusing on the second line of effort — a community needs assessment — Rivera provided an update on the goals and current status of three phases, each focused on a different group of key stakeholders. Phase 1 will explore the perspectives of MHS providers on FASD care and accessibility as well as delivery for all TRICARE beneficiaries throughout the MHS. The protocol for this phase has been approved, and interviews with primary care, internal medicine, pediatric, obstetrics/gynecology, and midwifery providers are set to begin soon. Subject matter experts helped create and pilot interview guides that are designed to capture a range of provider experiences related to screening, diagnosing, and management of possible or confirmed FASD through interviews that are around 30 to 45 minutes long.

Presently, the protocol is being modified for use in Phase 2 of the needs assessment, which is focused on parents and caregivers of children with FASD. FASD United will be heavily involved in developing interview guides that will be used with caregivers, Rivera noted. In Phase 3, the protocol will be modified to assess the needs of women who are pregnant or were recently pregnant.

### Screening tool use and perspectives

Colonel Paul Patterson, USUHS, presented new findings regarding military health care providers’ use and perspectives on the Survey of Wellbeing of Young Children (SWYC) tool. The SWYC is a validated, broad, free, quick, and easy-to-use tool that screens for developmental, behavioral, and family concerns, including food insecurity, family stress, and substance exposures. In June 2023, the Defense Health Agency began mandating the use of SWYC within the MHS at well-child checks taking place at 9, 18, 24, and 30 months.

Patterson noted that military children experience many of the same social determinants of health experienced by their civilian peers, but these can be further exacerbated by the frequent moves that occur in the military system. In light of the many factors that influence the well-being of military families, he said that there has not traditionally been a broad, comprehensive screening for all the factors that can influence these families. To better understand whether the SWYC is helpful in screening for social drivers of health in this context, Patterson’s team surveyed providers within the MHS.

The survey garnered responses from 95 clinicians from 55 hospitals, 91 of whom reported using the SWYC. Of the respondents, a little more than half said they use SWYC at every well-child visit, and just under half said that they supplement it with additional tools such as the Ages and Stages Questionnaire or Modified Checklist for Autism in Toddlers. Patterson pointed out that the fact that the SWYC is free means there is no need to budget for it every year, thus helping to reduce barriers to its use within MHS.

Respondents indicated that the SWYC gives them valuable insights into family dynamics, such as food insecurity and family stress, as well as children's behavior. They found the tool easy to use and integrate into their workflow. They also appreciate how the tool’s comprehensive approach extends beyond mere developmental assessment, offering a broader perspective.

The survey responses also surfaced a few downsides to using SWYC, including the fact that it was sometimes inconsistent. Because SWYC is a broad-based tool, it can tend to overidentify issues, Patterson noted. For example, it might indicate a risk in a child who perhaps does not have a true risk. Also, some providers felt they did not have the resources to deal with some of the screening results. For example, providers do not always know what to do if a family indicates they have a problem with food insecurity, domestic abuse, or substance abuse.

Looking forward, Patterson said that the researchers would like to use Genesis, the MHS electronic medical records system, to gather further data on SWYC usage. Patterson also pointed out that the data collected through SWYC could be useful for future research studies. In addition, the researchers, in conjunction with FASD United, have engaged the original SWYC developers on modernization of language employed for the substance use questions on the SWYC to make them more relevant and more appropriate for today’s families. Finally, they are continuing to collect provider feedback to better identify training gaps as well as resource deficiencies so that these can be addressed.

### Missed opportunities in FASD screening and diagnosis

Captain Meghan Lewis, Andrews Air Force Base and USUHS, presented the results of a survey that was administered at the Uniformed Services Academy of Family Physicians (USAFP) Annual Conference to find out what family physicians know about FASD. As family physicians take care of individuals from childhood to adulthood, including mother and baby, the goal was to determine how physicians screen for and diagnose FASD and how they educate their patients.

Researchers carried out the survey by adding 10 FASD knowledge and practice survey questions to the annual USAFP omnibus survey, which was sent to all conference attendees to anonymously complete online. Of the 612 people who attended the conference, 338 responded to the FASD questions. The clinicians who responded were split evenly between all of the different services. About 75% of them had graduated from residency within the last 10-12 years, which Lewis noted is relatively recent in terms of physician practice history.

The survey revealed a clear gap in physician training on FASD. Most respondents said they had received 0-1 hours of training on FASD during residency, and around 80% said they had received no FASD training since residency. Although most respondents answered at least one FASD knowledge question correctly, the majority were unable to answer more than this accurately. The number of training hours spent on FASD did not correlate with correctly answering the knowledge questions. The researchers also found that overall, screening for FASD is inconsistent due to various factors such as lack of time and/or resources. If screening is performed, provider use of diagnostic code for FASD is infrequent. Lewis said that providing tools for better access to appropriate screenings and support for positive results could help mitigate some of these issues.

Taken together, the study results identify several opportunities to enhance FASD awareness among uninformed family physicians, including education, screening, coding, and streamlining support for physicians to better assist their patients in this area, Lewis said. At the conference next year, the researchers plan to give a lecture to provide FASD education. They are also working on a manuscript for publication to help raise awareness and advocate for FASD training in family physician residency and/or medical education.

### FASD burden in the MHS

Elizabeth H. Lee, USUHS, reported preliminary findings from a study examining the burden of PAE, FAS, and related conditions in the MHS, which is part of the FASD Prevention and Clinical Guidelines Research initiative’s third line of effort. Her team used claims data from the MHS to look at PAE and FAS from 2016 to 2023, with a goal of better understanding the frequency of these diagnoses among military children, as well as the sociodemographic characteristics of diagnosed individuals.

Since FASD does not have its own ICD-10 code, the researchers used ICD-10 codes for PAE and FAS to identify relevant FASD cases for children 18 years old and under for all active-duty, guard, and reserve sponsors. For the seven years studied, they found a total of 1,479 children with a diagnosis of either PAE or FAS, 114 of whom had both diagnoses. Lee said that the data in her study suggest that as many as 145,000 children with FASD have been missed, with less than 1% of children with possible FASD being identified in the MHS.

The preliminary analysis also revealed that children with a PAE or FAS diagnosis were more often male and white. Based on the racial distribution of service members, Asians (5% of service members) and Blacks (17%) may be under-represented in this study while whites may be over-represented (70%), Lee said. The researchers also found that most of the children were diagnosed in a private (civilian) facility, with just 20% diagnosed at a military treatment facility or direct care. At the point of first diagnosis, 85% were classified as the service member’s child, which includes adopted and biological children.

Just over 40% of children with PAE or FAS had a parent in the Army, compared to 20% in the Navy and 30% in the Air Force. This roughly aligns with the distribution of military-connected children by their sponsor/parent’s service branch. Nearly 65% of these children had an active-duty parent who was of senior enlisted rank at the time of their diagnosis, which means that they may have been junior enlisted when the exposure first happened.

The mean age at PAE diagnosis was around 5 years old, with most children diagnosed before the age of 2. For FAS only, the mean age at diagnosis was nearly 10 years, with about 45% diagnosed between ages 12-18 years. Counterintuitively, about 60% of the 114 children who received both diagnoses were diagnosed with FAS first, and then with PAE roughly 1.5 years later.

Now that the researchers have a better idea of the burden of FASD in the military community, they plan to develop a broader case definition for FASD using American Association of Pediatrics codes, diagnostic guidelines, and subject matter expert inputs. They also aim to use their data to identify conditions that co-occur with FASD in the MHS. Finally, they plan to calculate annual adjusted rates of FASD and related conditions in the MHS. For the project’s fourth line of effort, which has not yet started, Lee said that the researchers will focus on understanding pathways through the MHS that children may take — from screening through to care and management for FASD — within the FASD continuum of care framework [17].

### Collaborative models for neurodevelopmental disorders

Eric M. Flake, USUHS, provided an update on progress for the FASD Prevention and Clinical Guidelines Research initiative’s fifth line of effort, which is focused on creating a tele-education and telehealth hub-and-spoke model across the MHS.

This effort blends three major activities: 1) tele-education for MHS clinical providers to raise awareness and support learning to improve providers’ ability to screen for and diagnose FASD; 2) improving neurodevelopmental diagnostic capabilities by using a virtual hub-and-spoke network of care that leverages the existing MHS telehealth infrastructure and supports diagnosis at the primary care level; and 3) piloting an at-home intervention to provide treatment techniques focused on self-regulation with support from parents, clinical staff, and navigators.

For context on these efforts, Flake highlighted statistics from the American Academy of Pediatrics indicating that approximately 40% of children younger than age 5 have some type of special health care needs, including neurodevelopmental or behavioral challenges (20-30% of those having developmental, behavioral personal social learning challenges, 10-15% with special health care needs, and 2-4% with severe disabilities). Recent years have seen a trend of increasing prevalence of children with developmental disabilities, and increasing care utilization and medication use for children with neurodevelopmental challenges and special needs. In the military, these neurodevelopmental and special needs fall within the exceptional family member program.

The research team is looking at using the tele-education model Extension for Community Healthcare Outcomes (ECHO) model to educate MHS providers. As part of this strategy, they participated in an ECHO program with the state of Alaska that military-connected health care providers were invited to join. The program included nine 2-hour FASD discussions. Of the 322 unique registrants, 75 had some military affiliation, and 36 indicated they provided care to the military community.

Defining diagnostic terminology is key to improving diagnostic capabilities. Flake pointed out that communities, families, and medical models often differ in the terms used, and terminologies sometimes change in various circumstances. However, there are very clear, specific neurodevelopmental disorders characterized by developmental deficits, deviance, or delay that produce impairments of personal, social, academic, or occupational functioning. These can be specified disorders, such as autism spectrum disorder or attention-deficit/hyperactivity disorder, with clearly defined diagnostic criteria, or they can be unspecified, which means that symptoms do not meet the full criteria for a specific neurodevelopmental disorder but still cause significant impairment. Flake said that in his experience, many families wish to have a specific identifier for their child’s condition, even if it may not be the best fit for them.

Flake also provided an update on the telehealth hub-and-spoke model under development to support FASD diagnoses and care in the MHS. Called the Hub and Spoke model The Supporting Toddlers to Adolescents Reach Success (STARS) Clinic, it is designed with a centralized "hub" that provides resources, training, and support to "spoke" locations, which include smaller clinics and bases. With a focus on strengths, the model provides pathways of support to recognize neurodiversity, support and celebrate self-advocacy, help families articulate and feel comfortable with having neurodiversity in their home, and identify the required supports. The model is designed to allow replication across systems and states and even globally. The researchers are working to establish a pilot hub in the Pacific Northwest.

To better understand existing FASD interventions, Flake worked with a team to perform a review of reviews examining behavioral interventions. The researchers studied nonpharmacological therapies delivered outside the school setting to determine the strength of the evidence for behavioral health interventions for children with FASD. After screening for eligibility, seven systematic reviews were included. The top interventions they identified included the Alert Program for Self-Regulation, the neurocognitive habilitation program, which includes psychoeducation and traumatic brain injury-related strategies, and Children’s Friendship Training, which includes group training to improve social skills and reduce problem behaviors. They are working now to interpret their findings, discuss the review's implications, strengths, and limitations, and then move on to publication.

### Discussion of scientific progress

During the discussion following the presentations, participants examined several key themes regarding the challenges faced in military and civilian healthcare systems, particularly in relation to data capture, stigma, and coding.

One issue participants raised relates to data access challenges for military data. Lee highlighted that while the current level of military data available through a centralized repository that includes health care data is commendable, it does not presently link to data such as military court decisions. While it does include more granular individual electronic health record data for care delivered at military facilities, it does not presently include this for individual care delivered at civilian facilities

Participants also pointed to stigma as a significant barrier to effective provider-patient communication, particularly regarding prenatal exposures. Michelle Kuhn, University of Washington, noted that providers often fear damaging relationships with their patients by addressing sensitive topics, which can lead to avoidance of necessary discussions. Lewis echoed this, stressing that a lack of information among providers perpetuates these barriers. Both experts pointed out that training in these sensitive conversations is crucial for overcoming stigma. Lewis noted that with proper training, health care providers could approach these issues more confidently and without judgment, increasing the utilization of available tools.

Several participants also highlighted the challenges associated with coding for FASD. Flake pointed out that underutilization of appropriate codes and vague definitions complicate accurate coding. Lewis emphasized that a general lack of education in billing and coding among military providers directly impacts the accuracy of documentation and secondarily delivery of service.

## Learning from living experiences

Jennifer Wisdahl, FASD United, introduced the living experience panel, which consisted of members from two military families who spoke about their experiences with FASD.

Aubrey Page has been in the military for 15 years, starting with four years in active duty as a Navy service warfare officer. After marrying an Army servicemember, both transitioned from active duty to the reserves. Now residing in Cincinnati, Ohio, they have embraced the role of foster parents. As foster parents, the couple began to see certain symptoms in the children they cared for that seemed to go beyond trauma. After attending FASD training, Page realized FASD could be contributing to disrupted placements for many foster families, and started providing FASD training to other families.

The Page family experienced deployment while having children in their home who qualified for the exceptional family member program (EFMP), a military program to identify family members who may have exceptional needs that would require them to be in a certain location. When a military member is up for orders, this need can be considered. She found out that although EFMP is considered in the Navy, the same was not true of the Army. When Page was deployed to Afghanistan, her husband chose to pause his reservist duties to prevent the possibility of both parents being deployed simultaneously. This decision was crucial in ensuring their son, who has autism, would not face the risk of institutionalization. She pointed out that the couple had decided to serve their community through foster care and adoption and also to serve their country through military service, but their experiences showed that the two types of service can be incompatible when the needs of a family conflict with the demands of military life.

Kathy White shared her perspective as a resource associate on the FASD United Navigator team and a Marine spouse. White and her husband have six children, one of whom has FASD. She said that even though she was very familiar with navigating TRICARE, having a child with FASD has presented significant challenges. Although her husband is now retired, she speculated that if they had a child with FASD while he was on active duty, relocating whenever the Marines sent them would have been extremely challenging for the child. She added that this situation could have also impacted her husband’s military career by hindering his chances for promotion.

The panelists identified stigma as an important issue for military families impacted by FASD. Page pointed out that alcohol is very normalized in both civilian and military culture and there are also a large number of unplanned pregnancies. At the same time, she said that people in the military may be hesitant to report that they have a substance use problem or that their child might have been exposed to alcohol in utero because of perceived consequences, such as career repercussions or the belief that having a mental health condition could result in the loss of a security clearance. She called for putting some protections in place so that people can feel safe, letting health professionals know about alcohol exposure while keeping their privacy and getting appropriate care and support for their child and family. She also added that prevention and treatment for substance use disorders is an important part of this issue, as well.

The panelists also talked about some of the supports that are available for military families. For example, the Navy’s Fleet and Family Support Center can help parents get more information, or get connected to the right place to learn more about brain-based disabilities and gain perspective. White pointed to the Buckley spouses group, which provides support and connections when new families move into an on-base neighborhood. Replicating this for off-base families could be beneficial, she suggested. In addition, the panelists pointed out the need to better connect children’s hospitals in the community with on-base care, since children with more complex needs may require services at the local children’s hospital.

## Strategies for improving support for families

Rivera introduced the meeting’s working session, which sought to elicit the perspectives of various groups to identify opportunities to improve support for families dealing with FASD. To set the stage for the working session, Rivera defined some key terms. For the purposes of the discussion, family-directed care was defined as care focused on educating the parent or caregiver with the goal of better enabling them to manage their child's FASD symptoms, while health care-recommended support or interventions include any support or intervention that is recommended by a clinician or medical provider to help manage FASD symptoms.

Rivera pointed out that being associated with the military brings many positive features, such as a close network of support from other families, as well as some unique challenges. For many military-connected children, frequent relocation means frequent changing of schools, social networks, support systems, and medical care. In addition, the added stress of a parent being deployed or away from home — along with associated worries that might be experienced by the parent who cares for the family during a deployment — can affect both physical and mental health for the whole family.

The working sessions focused on the perspectives and priorities of different communities, including families and caregivers, health care providers, and advocates and researchers. After engaging in separate discussions, representatives from each group shared some key take-aways that surfaced during their conversations. Overall, the report-outs highlighted the need for a multi-layered, informed approach to supporting FASD families in the military community.

### Families and caregivers

Families and caregivers voiced the critical need for support and connections. They emphasized that families require strong support networks, including support from other FASD caregivers and community members who understand the challenges without judgment. This "village" concept could help support both practical needs and emotional resilience. FASD-informed respite care options and access to specialized mental health services for all family members are also critical.

Participants in this group also said that families need advocacy assistance to help them access FASD-specific resources, which can often be hampered by long waitlists, financial strains, and lack of insurance coverage for FASD-related care. Many parents also need help coordinating care with multiple providers and accessing educational support, for example through IEP advocacy.

As noted throughout the workshop, frequent relocations bring unique challenges for military families. To address the pressing need to ensure continuity of care, participants suggested that tools akin to a care “passport” could provide a central resource for key information about the child’s needs, which can then be shared with new health care providers or schools. Participants emphasized that TRICARE and other military support programs need to ensure continuity of FASD care regardless of where families are stationed. For example, TRICARE remote could improve access to telehealth programs like SNACS and Triumph, which deliver scalable family support using virtual technology.

For military families living off base, customized support options are needed to reduce gaps in care. Finally, participants said that transition-age planning is also vital, especially for older children approaching adulthood, to ensure they maintain the support they need in new environments.

### Health care providers

Participants in this group stressed the need for FASD-specific training and sensitivity among health care providers. A lack of specialized training in FASD can lead to inappropriate or even harmful approaches. For example, they noted that trauma-informed care, focused on understanding developmental gaps, can better support these children than behaviorist methods that do not align with the needs of individuals with FASD.

Clinicians can help to reduce the stress of managing complex care needs by providing caregivers with written and visual materials that reinforce provider instructions. Warm handoffs, where providers refer families to open, available specialists, also reduce barriers and improve care access. For instance, participants suggested that FASD-informed coordinators could streamline care and act as a central support figure to ensure families have a professional ally in the healthcare system.

Participants emphasized that it is essential for health care providers to move from only diagnosing or screening for FASD to providing actionable next steps and continuity in care. Recognizing the ongoing nature of FASD care can help providers focus on long-term, adaptive strategies rather than isolated interventions.

### Advocates and researchers

Reliable data on FASD prevalence and family needs plays an essential role in justifying research and intervention funding and shaping effective policies. Recognizing this, participants said that data could be enhanced by standardizing FASD diagnostic codes to streamline data collection and improve funding and intervention efforts.

They also emphasized the need to reduce stigma by promoting education among health care providers, educators, and the general public. Increasing awareness can make it easier for families to access care and normalize seeking accommodations without judgment. For active-duty personnel or families concerned about the impact of FASD on career opportunities, addressing potential stigmas within the military system is critical. Participants stressed that seeking support for FASD should not limit a person’s job prospects, and added that TRICARE can play a role in ensuring that FASD-informed care does not create barriers to career advancement. Finally, participants suggested that normalizing the need for accommodations, advocating for FASD-informed care, and addressing stigmas that may affect caregivers — especially in military settings — could improve access to resources and make it less daunting for families to seek help.

## Data Availability

All data is contained within the workshop report

## Appendix A

**Table 1:** Workshop attendees

**Organization Type**	**Organization**	**Participant**
Government	Centers for Disease Control and Prevention	Elizabeth Parra Dang, MPH
	Health Resources and Services Administration	Sonsy Fermín, MSW, LICSW
	Uniformed Services University of the Health Sciences	Paul Crawford, MD
		Barbara Fuhrman, PhD
		Lynette Hamlin, PhD, CNM, Co-PI
		Tracey Koehlmoos, PhD, MHA, PI
		Elizabeth Lee, DrPH, MPH, Co-I
		Meghan Lewis, MD, FACS
		Jeanmarie Rey, Co-I
	Walter Reed National Military Medical Center	LTC Paul Patterson, LTC, MD, PhD
Non-profit organizations	Families Rising	Barb Clark
	FASCETS	Nathalie Brassard, BSc
	FASD - North Dakota	Carl Young, MS
	FASD Collaborative Project	Angelle Gremillion
	FASD Focus NW	Michelle Kuhn, PhD
	FASD Network of Northern California	Rachel Sing
	FASD Network of Southern California	Annette Kunzman
		Janis Reid, MSW, LCSW
	FASD United	Kate Boyce, JD
		Sarah Brown, MPH
		Susan Carlson, JD
		Tom Donaldson, BA, Co-I
		Kristen Eriksen, RN
		Andy Kachor, BFA
		Musa Kamra
		Chris Melfi, BA
		Sophie Sissi, MA
		Kathy White
		Jennifer Wisdahl, BA
	Formed Families Forward	Kelly Henderson, BA
		Stacia Stribling, PhD
	Indiana Alliance on Prenatal Substance Exposure	Maria Casbon
	Institute for Health and Recovery, Inc.	Reuben Kittrell
	KA FASD	Robin Burgamy
	Lauola Pono Foundation	Michael Anderson
	NCFASD Informed, Inc.	Terry Henson
		Kathy Hotelling, PhD, ABPP
	Papillion Center for FASD	Chris Troutt, LMFT
	Proof Alliance	Julia Conkel-Ziebell, PhD, LP
		Kendra Gludt, MPH
	Proof Alliance NC	Lauren Borchert, BS
	Southwestern Mental Health	Luke Comeau, MA
	Texas FASD Network	Julia Rivera, Col USAF Judge Advocate General
	The FASD Collaborative	Emily Rusnak, PhD, CCC-SLP
	The Florida Center	Tamra Cajo
	The Florida Center for Early Childhood	Jennifer Werden, MSW
	Violence Intervention Program, Community Mental Health Center	Michele Walker-Bauer, PhD
Companies and consultancies	Brain First Family Center	Ryan Jolly, MSN, APRN, PMHNP-BC
	Guided by Guida	Guida Brown, MA
	Pienovi Healthcare, LLC	Sil Pienovi
	United States Drug Testing Laboratory, Inc.	Aileen Baldwin, PhD, MPH
Patients and families	Self/no affiliation	Angela Freeman
		Liz McHugh
		Stephanie McNerney
		Aubrey Page
	Dream Acres	Cliff Meinhardt
		Kathryn Meinhardt
Academic research and health care organizations	Baylor College of Medicine	Roger Zoorob, FAAFP
	Boston Children's Hospital	Olivia Weeks, PhD
	Healthy Native Nation, UC San Diego	Annika Montag, PhD
	Henry M. Jackson Center for the Advancement of Military Medicine, supporting the Center for Health Services Research at USUHS	Madison Cirillo, MPH
		Eric Flake, COL (Ret.)
		Cathaleen Madsen, PhD
		Sharon Pritchett, MPH
		Ilse Rivera, MPH
		Zoe Solomon, MPH
		Elta Liang, MPH
	Louisiana's University Center for Excellence in Developmental Disabilities, Human Development Center	Krista James, Med, PhD
	Seattle Children's Research Institute	Carmichael Olson, PhD
	Syracuse University	Sarah Hamersma, PhD
	Texas A&M School of Medicine	Amanda Mahnke, PhD
		Siara Rouzer, PhD
	University of Arkansas Partners for Inclusive Communities	Leslie Whitefield, APRN
		Tiffany Lepard, MS
		Natalie Welcher, PSSP-SLP
	University of New Mexico Center for Development and Disability	Marcia Moriarta, PsyD
	University of Rochester	Lynn Cole, RN
		Carson Turnbull, MA
	University of Rochester Mount Hope Family Center	Julianne Myers, PhD
		Christie Petrenko, PhD
	University of Washington	Tracy Jirikowic, OTR/L
Organization not specified		Sam Elkins

## Abbreviations

FASD: Fetal alcohol spectrum disorders
USUHS: Uniformed Services University of the Health Sciences
DoD: Department of Defense
MHS: Military Health System
CHSR: Center for Health Services Research
HSR: Health Services Research
FAS: Fetal alcohol syndrome
PAE: Prenatal alcohol exposure
CDC: Centers for Disease Control and Prevention
ND-PAE: Neurobehavioral disorder associated with prenatal alcohol exposure
IEP: Individualized education plan
ECHO: Extension for community healthcare outcomes
FMF: Families Moving Forward
SNACS: Specialized Neurodevelopmental Assessment and Consultation Service
SWYC: Survey of Wellbeing of Young Children
USAFP: Uniformed Services Academy of Family Physicians
STARS: Supporting Toddlers to Adolescents Reach Success
EFMP: Exceptional family member program
